# Investigation of Voids Characteristics in an Asphalt Mixture Exposed to Salt Erosion Based on CT Images

**DOI:** 10.3390/ma12223774

**Published:** 2019-11-17

**Authors:** Rui Xiong, Wenyu Jiang, Fa Yang, Kehong Li, Bowen Guan, Hua Zhao

**Affiliations:** 1 School of Materials Science and Engineering, Chang’an University, Xi’an 710061, China; 2 Engineering Research Center of Transportation Materials of Ministry of Education, Chang’an University, Xi’an 710061, China; 3 Yunnan Communications Investment & Construction Group Co., Ltd, Kunming 650228, China; 4 School of Civil Engineering and Architecture, Nanchang University, Nanchang 330031, China

**Keywords:** asphalt mixture, salt erosion, CT technology, image segmentation, voids characteristics, Correlation analysis

## Abstract

The performance of an asphalt mixture will deteriorate under the condition of salt erosion, but there are different opinions on the mechanism of deterioration. Few studies have focused on the relation between the change of void characteristics and performance deterioration of an asphalt mixture exposed to salt erosion. To explore the relation between the air voids characteristics of an asphalt mixture and mechanical damage under salt erosion, the mechanical damage in an asphalt mixture was measured by splitting strength. The asphalt mixture specimens, immersion solutions, asphalt mortar, and aggregate were scanned with CT technology. To segment the voids, the Otsu method was used over asphalt mortar and solution range of CT values. A three-dimensional reconstruction of the CT image was performed with Mimics 20 software to calculate the asphalt mixture’s void characteristics. On this basis, the relationships between the change in void characteristics and splitting strength were analyzed. The results showed that the ideal calculated void fraction can be obtained by threshold segmentation of the image void/asphalt mortar interface with the local CT value Otsu method. Under the salt corrosion environment, the increase of open voids of an asphalt mixture is linearly correlated with the decrease of splitting strength, while salts’ crystallization in the open voids produces crystallization pressure, accelerating the volume growth of open voids. The early damage of an asphalt mixture suffered from the salt may be mainly physical damage. These results can provide a useful reference for the performance of damage research on asphalt mixtures in salt enrichment areas.

## 1. Introduction

In coastal areas with frequent marine fog and high salinity and humidity, inland saline lakes, saline soil areas, and northern areas where chlorine salts, such as road deicing salts and snow-melting agents, are used widely, salt erosion compromises a pavement’s performance [1,2]. Most research to date largely has studied salt erosion’s influence on asphalt mixture performance according to changes in splitting strength, rutting factor, and other mechanical properties before and after exposure to different salt erosion conditions (dry–wet cycle, freeze–thaw cycle, etc.) and different salts (NaCl, Mg_2_Cl, Ca_2_Cl, Na_2_SO_4_, etc.) [3,4,5]. Because the macro-mechanical test index is based on repeated tests, asphalt mixture performance is not equal to the macro-physical index [6]. With the deepening of the research, various erosion deterioration mechanisms have been put forward.

Xiong R et al. used the rutting test to prove that the chlorine salt solution would cause high-temperature deformation ability of an asphalt mixture to decrease and the NaCl crystallization would act as a lubricant between aggregates and reduce the internal friction in asphalt mixture [7]. Wu Z M et al. found that NaCl crystallization pressure can cause internal damage (micro-cracking) of an asphalt mixture by low-temperature trabecular bending tests, XRD and SEM, and thereby mechanical strength of an asphalt mixture is decreased [8]. Ma Q Y and Qing K et al. proved that the surface tension of the chloride salt solution was greater than the surface tension of asphalt and the chloride salt solution was more likely to invade into the joint of the asphalt and aggregate, resulting in the adhesion decrease between asphalt and aggregate [9,10]. Zhou J Z et al. believed that the erosion of chloride ions accelerated the aging of the asphalt in the mixture, thereby degrading the performance of the asphalt mixture [11]. Cha X D et al. used NaCl and CaCl_2_ to carry out the erosion tests of an asphalt mixture, which proved that Na+ promoted the emulsification of asphalt and reacted with asphalt to form a highly unstable adsorption layer, causing the asphalt mixture strength to decrease [12]. However, up to now, few studies have explained the deterioration mechanism of salt erosion to asphalt mixture from the perspective of voids structure. 

CT is a non-destructive testing method based on imaging different X-ray absorption coefficients of different substances. The first application of X-ray CT technology to study the internal composition of an asphalt mixture is the SIMAP program (1998) jointly implemented by the US Federal Highway Administration and Turner Highway Research Center (TFHRC) [13]. Subsequently, Masad E and Wang L B et al. have published relevant research results [14,15,16,17,18,19].

The void distribution in asphalt mixture is the key factor that affects their mechanical properties [20]. Nowadays, the analysis of voids by CT scanning and image processing technology is popular in the meso-research into asphalt mixtures [21,22]. Yu W S and Masad et al.studied asphalt mixture void distribution based on CT scanning technology [23]. Hu J et al. improved the adaptive threshold segmentation algorithm based on the annular region to study voids and aggregates’ shape and distribution [24]. Gao J et al. proposed a three-stage method to analyze the law of void distribution, and based on this, established a method to predict asphalt mixture void ratio [25]. He J and Zhou X L et al. analyzed the fractal dimension of mixture voids based on CT images [26,27,28]. CT is increasingly mature in the microscopic analysis of an asphalt mixture. In view of this, the paper used two kinds of erosion methods to carry out salt erosion damage on AC asphalt mixture, and tested its mechanical properties degradation trend under five salt concentration gradients along with four erosion ages. The CT image analysis results were supplemented by SEM microscopic test methods to explain the deterioration mechanism of an asphalt mixture exposed to salt erosion. The results can provide a useful reference for research on asphalt mixture degraded performance in salt enrichment areas.

## 2. Experiment

### 2.1. Materials and mixture design

#### 2.1.1. Asphalt

The SK-90# matrix asphalt whose penetration is between 80~100 (0.1 mm) was used for all experiments. Its main technical properties are shown in Table 1.

#### 2.1.2. Aggregate and corrosive salinity

All of the coarse and fine aggregates used in the test were made of basalt, and the filler was ground limestone ore powder. The mineral aggregate’s gradation was AC-13, and each grade of aggregate’s passing rate is shown in Table 2. The asphalt mixture’s optimum asphalt–stone ratio is 4.78%, as determined by the standard Marshall test. Industrial salt was selected for the test. The technical indices are shown in Table 3.

### 2.2. Methods

The asphalt mixture specimens were prepared by the standard Marshall test method according to the “Test Rules for Asphalt and Asphalt Mixture in Highway Engineering” (JTG E-20-2011). The specimens’ initial void fraction was measured by the surface drying method. Eleven groups of molded dry specimens were used to meet the specifications’ requirements, and were labeled AC(0)~AC(10). There were four specimens in the blank group, and the remainder included 16. All immersed specimens had to be saturated with water before the test. The specific test steps are as follows: place the specimen in a box filled with clear water with the water surface 2 cm higher than the top surface of the specimen and place the box in the vacuum instrument at a negative pressure of 3.7 + 0.3 kPa for 20 min. After vacuum treatment, the salt water erosion test was carried out in the test environment shown in Table 4.

The specific laboratory test environments were as follows: 

The salt immersion environment was as follows: the sample was immersed horizontally at 40 °C. During the test, the test box is covered to prevent test error attributable to water evaporation.

The drying and wetting cycle environment was a 24 h cycle (soaking for 12 h, drying for 12 h); the soaking condition were the same as above, while the drying condition entailed removing the sample horizontally and placing it in an oven set at 40 °C.

According to the “Test Rules for Asphalt and Asphalt Mixture in Highway Engineering” (JTG E20-2011), the freeze–thaw splitting test for an asphalt mixture was used to test the AC (0) specimens and other specimens’ initial splitting strength at 7, 14, 21, and 28 days of age.

### 2.3. CT Scanning Test

**Figure 1:**
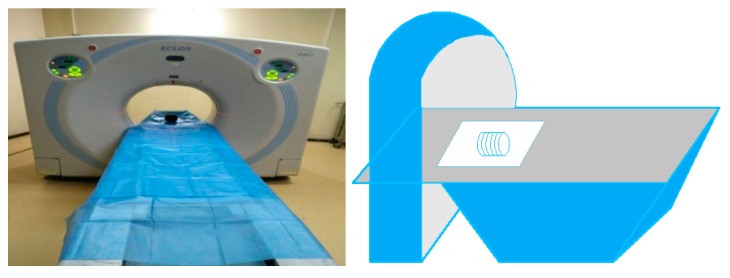
CT test stand.

The CT equipment used in this experiment was the Hitachi ECLOS.16 row spiral CT (HITACHI, Zun’yi, Guizhou Province, China), the CT test stand is shown in Figure 1, the scanning parameters of which are shown in Table 5.

**Figure 2:**
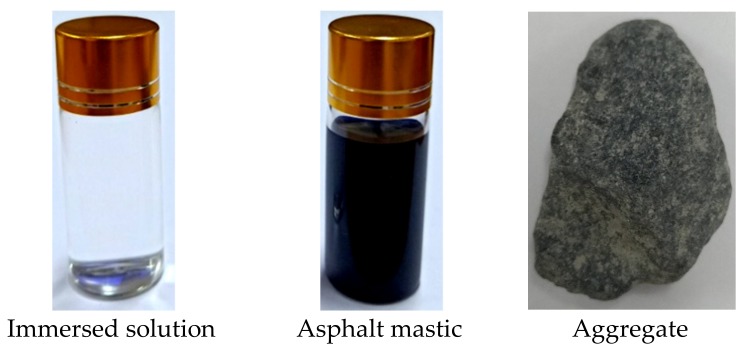
Asphalt scanning sample.

The initial asphalt mortar, aggregate, distilled water, and a 20% salt solution, respectively, were put in small glass bottles (22 × 60 mm), and their corresponding CT values were obtained by CT scanning. The scanning sample is shown in Figure 2.

**Figure 3:**
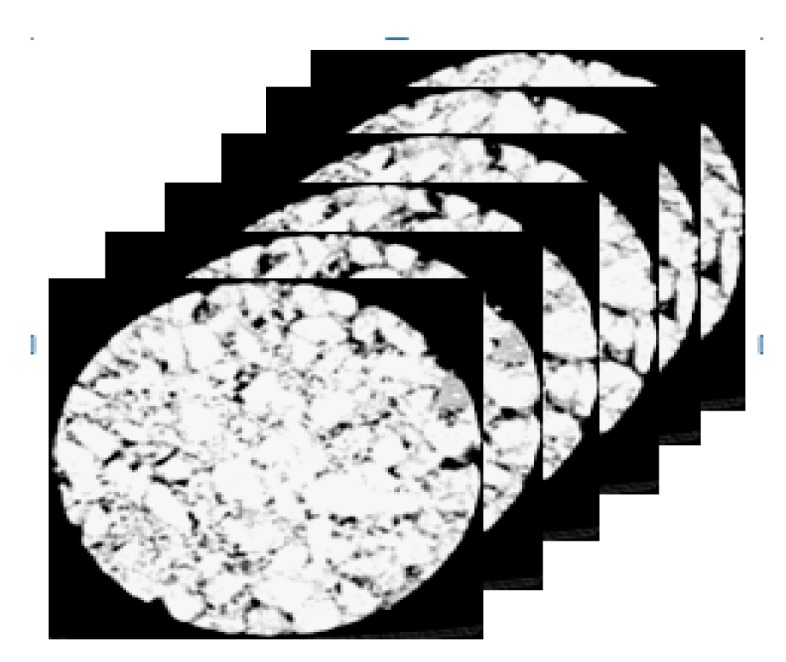
Scanned image overlay.

Four standard Marshall specimens were selected and referred to as AC1, AC2, AC3, and AC4. The test environment is shown in Table 6. As CT is a non-destructive testing method, the same specimens were scanned continuously at 0, 7, 14, 21, and 28 days of age. Before scanning, samples were removed from the water and salt water, wiped with a towel to remove their surface moisture, and then subjected to CT scanning. The CT-scanned specimens’ test environment is shown in Table 6. The conditions for the soaking and drying-wetting cycle are the same as those in Table 4. Each specimen was scanned 42 times. Given that compaction affected the specimens’ upper and lower ends greatly, 40 middle CT images were selected for experimental analysis, and the specimens’ original CT images were obtained. Some of the scanned images are superimposed, as shown in Figure 3.

## 3. Image Processing

**Figure 4:**
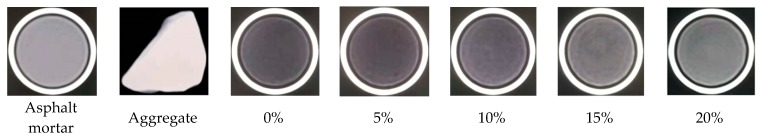
CT Images of Each Part.

Because this paper focused on voids, image processing focused on the segmentation of the asphalt mortar/void interface. Because the specimens were immersed, there was water (brine) at the interface between the asphalt mortar and the specimens’ open voids that affect the image processing results. The CT images of the asphalt mortar, aggregate, and salt solution at various concentrations are shown in Figure 4, and the CT values are presented in Table 7.

From Table 7, it can be seen that the asphalt mortar and solutions’ CT values were similar, such that the gray histogram of the asphalt mixture’s CT image does not show obvious double peaks, and thus, the double peaks method is not suitable for segmentation. Therefore, in this paper, the gap and asphalt mortar were separated by calculating the T value using the Otsu method over the range of CT values. The specific steps were as follows: Over the range of CT values, the asphalt mortar’s histogram void and CT value, the void’s segmentation threshold (i.e., target), and the asphalt mortar were recorded as T, and the proportion of the void pixel points to the pixel points in the segmentation area of the CT value histogram was recorded as ω0, while the average CT value was μ0. The proportion of pitch mortar pixels in the CT value histogram segmentation area was ω1, and the mean CT value was μ1. The segmented area’s total mean CT value was recorded as μ and the variance between classes was recorded as g.

The total number of pixels in the segmentation area was M, the number of pixels with a CT value less than the threshold T was N0, and the number of pixels with a CT value greater than the threshold T was N1.

(1) ω0=N0/M

(2) ω1=N1/M

(3) N0+N1=M

(4) ω0+ω1=1

(5) μ=ω0·μ0+ω1·μ1

(6) $$g=\omega 0\cdot {\left(\mu 0-\mu \right)}^{2}+\omega 1\cdot {\left(\mu 1-\mu \right)}^{2}$$

The equivalent formula is obtained by substituting Equation (5) into Equation (6):

(7) $$g=\omega 0\cdot \omega 1\cdot {\left(\mu 0-\mu 1\right)}^{2}$$

The threshold T, which maximizes the variance g between classes, was obtained by the traversal method.

**Figure 5:**
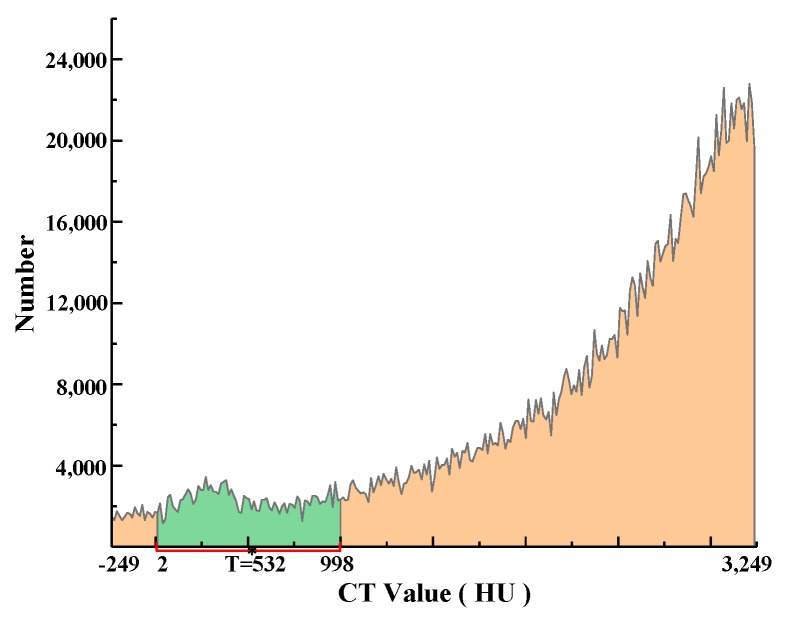
Image Segmentation Schematic Diagram.

**Figure 6:**
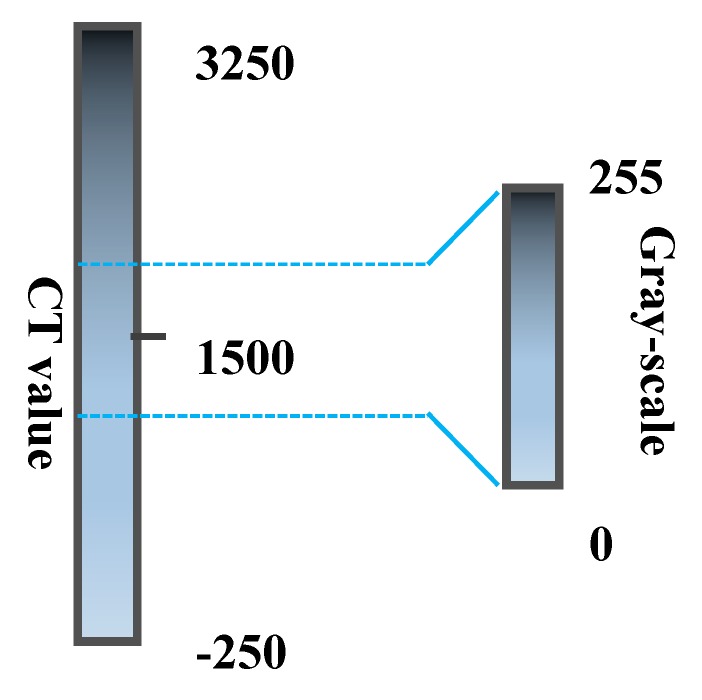
Relationship between the Gray and CT Values.

The number of CT values in the sample CT image was counted, and a histogram of CT values was drawn. Equation (7) was used to calculate the CT values in the range of 2HU-998HU, and T = 532 HU was obtained. The segmentation diagram is shown in Figure 5. To verify this method’s applicability, the gray value of the CT image was calculated by the Otsu method at the same time, and the threshold T1 = 74 was obtained. The value of T1 was converted to 766 HU, which was recorded as T1. The schematic diagram of the gray value and CT value conversion is shown in Figure 6. When the CT value exceeds 3250 HU, the gray value is 255, and when the CT value is less than −250 HU, the gray value is 0. When the CT value is between −250 HU and 3250 HU, the conversion formula between the CT and gray values is as follows:

(8) B×3500÷255−250=A

B-gray value; A-CT value.

**Figure 7:**
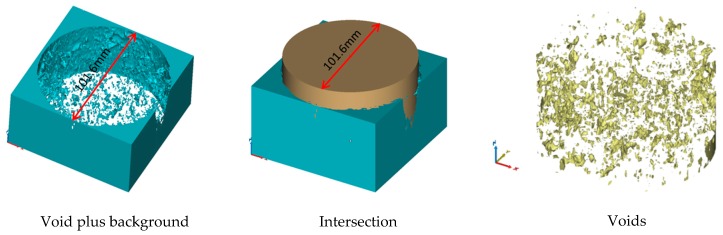
Diagram of Void Calculation.

**Figure 8:**
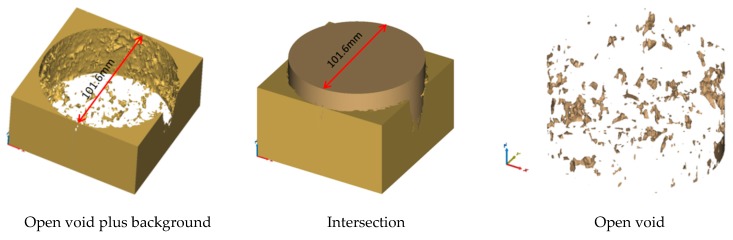
Schematic Diagram of Open Void Calculation.

Based on the three-dimensional reconstruction software (Mimics 20), the T and T1 values were set as image segmentation parameters for the three-dimensional reconstruction of the CT images. The sketches to calculate the voids and openings are shown in Figure 7 and Figure 8. The void volume, number, and fraction were calculated. The open voids’ volume, number, and void fraction were calculated by local growth.

After the three-dimensional reconstruction with T, the specimens’ T1 segmentation thresholds and void values were calculated and compared with those measured by the surface drying method. The results are shown in Table 8. The voidage value obtained from the test was taken as the true voidage value, and the error analysis of the voidage value calculated was carried out. The results of the relative error analysis are shown in Table 9.

(9) D=|X−x|

(10) $$\mathrm{Er}=\frac{D}{\left|X\right|}$$

X-true voidage value; x-calculated voidage value; D-absolute error; Er-relative error

Table 8 and Table 9 show that the voidage calculated by the local CT value Otsu method is closer to the voidage value measured. This shows that this image processing method can obtain the mixture’s ideal void fraction under the condition of solution immersion. Therefore, this method was used for image segmentation in all of the gap analyses in this paper.

## 4. Results and Discussion

### 4.1. Analysis of Splitting Strength Results

**Figure 9:**
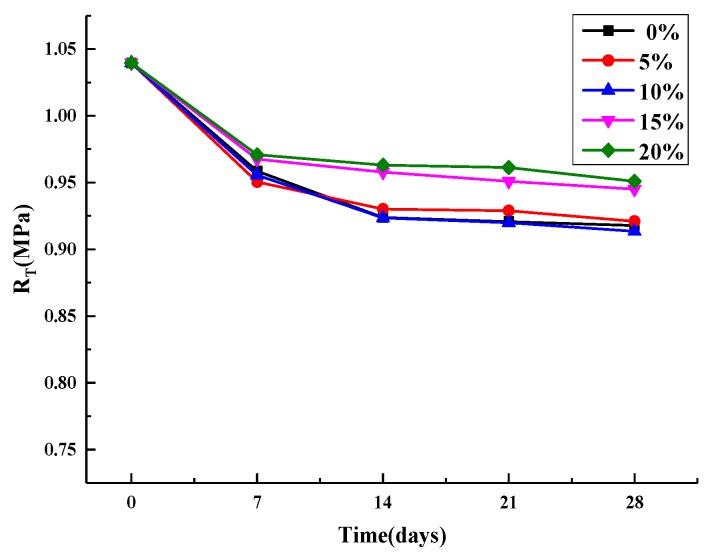
Soaking directly splitting strength.

**Figure 10:**
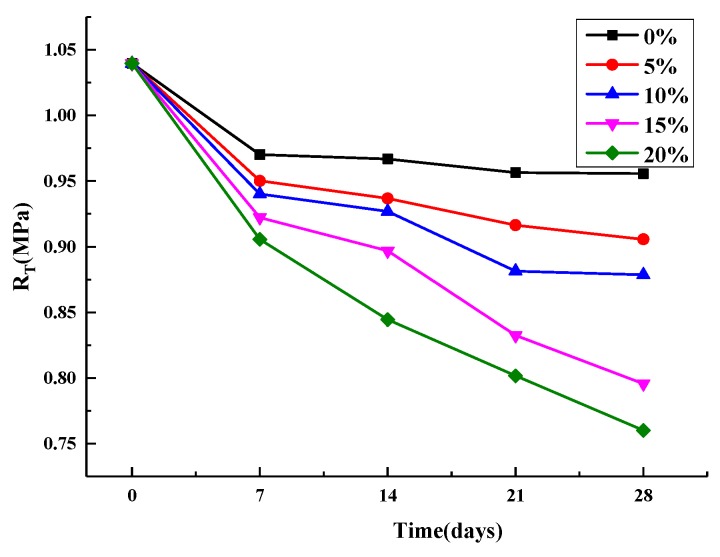
Dry and wet cycle splitting strength.

**Figure 11:**
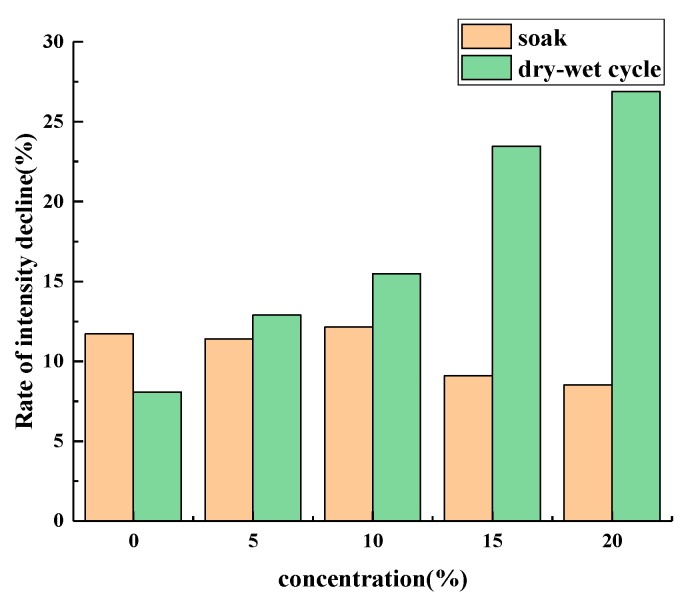
Damage degree of splitting strength at 28 days.

The results of the splitting strength and 28 d splitting strength loss are shown in Figure 9, Figure 10 and Figure 11.

Figure 9 shows the change trend of splitting strength of an asphalt mixture with the increase of age under the condition of direct immersion in five salt concentration (including 0%). It can be seen that the damage trend of salt erosion is similar to that of water erosion (0% salt concentration) under this erosion method. As the age increased, the strength of the specimens soaked in clear water decreased most rapidly at 28 days, by 11.7%, while the strength of the specimens soaked in 20% brine decreased most slowly, by 8.5%. Given that there is salt crystallization in 20% brine-immersed specimens that fills small voids, the splitting strength increased, which counteracts some of the damaged by water [29].

Figure 10 shows that the splitting strength decreased with increased age under the condition of wet-dry cycling. The higher the salt concentration is, the faster the rate of decline becomes. After 28 days, the specimens’ strength decreased most slowly under the dry–wet cycle with clean water, 8.1%, and 26.9% under the dry–wet cycle with 20% brine. This is because under dry–wet cycling conditions, salt crystallization produces expansion pressure, and the higher the salt concentration, the greater that pressure, and the greater the damage to the asphalt mixture.

The results suggest that the strength of the samples under the dry–wet cycling with the salt solution decreased significantly faster than that of the samples immersed directly, as shown in Figure 11. This may indicate that there are more changes that have taken place in the asphalt mixture during the salt water drying and wetting cycle. In order to analyze the deterioration mechanism of an asphalt mixture performance under the condition of salt erosion from the microscopic point of view, the mixture’s void evolution characteristics under the maximum and minimum damage conditions were investigated by CT scanning (i.e., 0%, 20% salt water concentration immersion and dry–wet cycle).

### 4.2. Calculating the Evolution Value of Void Characteristics

Evolution values for the AC1, AC2, AC3, and AC4 specimens’ void characteristics were calculated as shown in Table 10, Table 11, Table 12 and Table 13.

Table 10, Table 11, Table 12 and Table 13 shows the variation of void characteristics of an asphalt mixture under different erosion conditions. It can be seen that the voidage and open voidage of an asphalt mixture in all erosion environments increases with the erosion age, while the number of voids and open voids decreases. The influence degree of each erosion environment on void ratio and opening void ratio is similar to that of splitting strength. 

### 4.3. Relevance between Open Void and Splitting Strength

**Figure 12:**
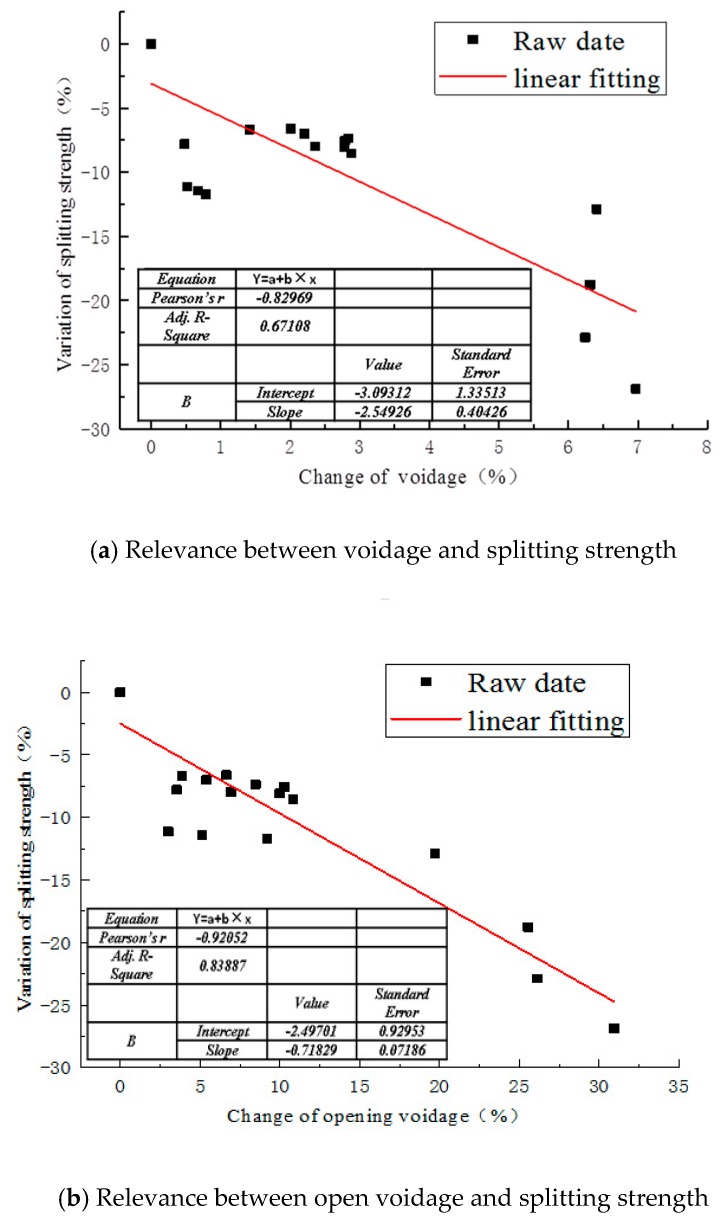
Relevance between void and splitting strength.

The experiments demonstrate that compared with the change of total voids, the loss of splitting strength is highly correlated with the change of open void fraction, as illustrated in Figure 12. This may indicate that the open voids are most likely to be damaged when the asphalt mixture is in an erosive environment, leading to the performance deterioration of an asphalt mixture. 

**Figure 13:**
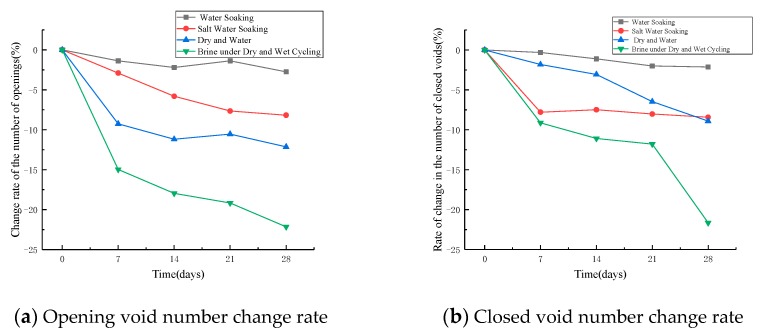
Change rate of voids in asphalt mixture.

Figure 13 shows the change in the number of open voids under four erosion conditions. As shown in Figure 13a, the number of opening voids of an asphalt mixture in four erosion conditions decreases with the increase of erosion age. The decrease rate of the number of open voids in asphalt mixture with salt dry–wet cycle is much higher than that under the other three conditions. Under the condition of direct soaking, the number of open voids of an asphalt mixture decreases slowly with the increase of age. Under the condition of dry–wet cycle, the number of open voids decreased sharply in the early stage and tended to be flat in the later stage. The decrease rate of the number of open voids at 7 d of erosion can reach 67.6% of total decrease rate at 28 d under the condition of salt water dry–wet cycle. 

It can be seen from Figure 13b that the number of closed voids of an asphalt mixture in four erosion environments also decreases with the increase of erosion age. Under the condition of direct soaking, the decrease of the number of closed voids is concentrated in the early stage of erosion, and tends to be gentle in the later stage. However, under the condition of dry–wet cycles, the number of closed voids in asphalt mixture tends to decrease rapidly in the later stage of erosion. These results may indicate that the erosion from salt water to the asphalt mixture’s void structure is from the outside to the inside.

**Figure 14:**
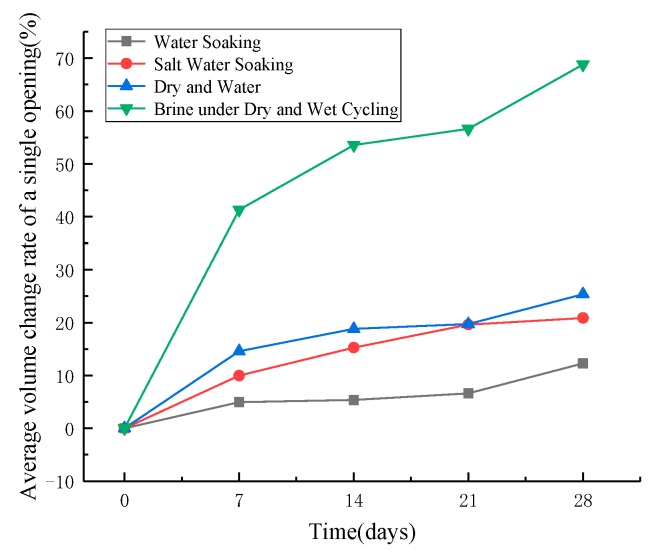
Volume change of single opening void.

Figure 14 shows the change of average void volume of an asphalt mixture. It can be seen that the average open void volume of an asphalt mixture increases with the increase of erosion age. Except for the salt water dry–wet cycle, the average open void volume under the other three erosion conditions does not increase more than 25.3% at 28 d. At 28 d, the average volume of the open void of the asphalt mixture increased by 68.8% compared with that of the asphalt mixture without salt erosion. 

**Figure 15:**
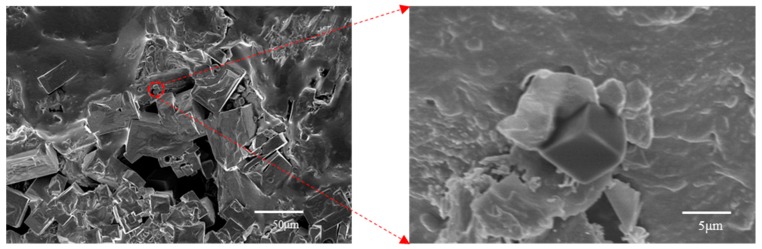
Salt crystal attached to pore wall.

**Figure 16:**
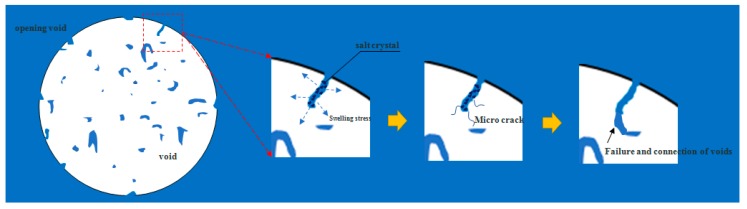
Volume change of single opening void.

On the whole, the results of void characteristics may indicate that salt crystallizes on the pore wall of the opening space in an asphalt mixture under the condition of dry and wet salt water (Figure 15), resulting in expansion stress, which leads to the generation of microcracks on the pore wall. Micro cracks are the beginning of failure [30,31]. With the increase of erosion age, the opening voids will run through other voids from the outside to the inside, resulting in the decrease of the number of voids and the increase of opening voids, as illustrated in Figure 16. As mentioned in Section 4.3, the increase of opening voids directly leads to the deterioration of an asphalt mixture performance. Meanwhile, the early salt erosion deterioration of an asphalt mixture performance is due to the expansion stress produced by the crystallization of salt on the pore wall, which is likely to lead to the expansion of the opening voids to the interior of an asphalt mixture, and then connect with other voids, which aggravates the deterioration of an asphalt mixture performance. In high-humidity coastal areas, the early damage of an asphalt mixture exposed to salt may be mainly physical damage.

## 5. Findings and Conclusions

The findings may be summarized as follows:

- In this paper, the local CT value Otsu method is proposed to conduct threshold segmentation of CT images of an asphalt mixture under the condition of solution immersion. By using this threshold to reconstruct three-dimensional specimens, the ideal void structure can be obtained.
- Physical factors play a dominant role in the asphalt mixture’s salt damage. The mixture’s strength decreases more slowly under the condition of direct brine immersion than that of direct water immersion. However, the splitting strength of an asphalt mixture decreases significantly with an increase in salt concentration under the condition of a salt dry–wet cycle.
- The change of open porosity in a solution immersion environment is an important factor affecting the fracture strength of splitting. The main reason of the splitting strength failure in the salt dry–wet cycle is that salt crystallizes in the open voids and the crystallization pressure increases the open voids, connects with the surrounding open voids and decreases their number subsequently.

## 6. Research Prospects

- At present, for the damage mechanism of an asphalt mixture under salt erosion environment, this paper studies it just in a short period, and will further simulate half a year or even longer salt erosion ages in the next work.
- It is important to realize the virtual test of an asphalt mixture and the prediction of pavement service life under salt erosion environment, which is also the next step of the research.

## Figures and Tables

**Table 1:** Technical Properties of SK-90# Asphalt Binder.

Material	Test Items	Unit	Value	Specification
Matrix asphalt binder	Penetration at 25 °C	0.1 mm	86	ASTM D5-97
	Ductility at 15 °C	cm	182	ASTM D113-99
	Softening point	°C	47.0	ASTM D36-06
	Wax content	%	1.75	ASTM D3344-90
	Flash point	°C	304	ASTM D92-02
	Specific gravity	Non	1.030	ASTM D70-76
RTFO binder *	Mass loss	%	0.15	ASTM D2872-04
	Penetration ratio at 25 °C	%	60.5	ASTM D5-97
	Ductility at 10 °C	cm	9.8	ASTM D113-99

* Rolling thin film oven (RTFO) aged, according to ASTM D2872-04.

**Table 2:** Aggregate Gradation for AC-13 Mixture Design.

Sieve Size (mm)	16	13.20	9.50	4.75	2.36	1.18	0.60	0.30	0.15	0.08
Percent passing (wt. %)	100	95.50	72.00	47.10	33.40	23.40	15.20	8.70	7.30	5.20
Required passing range (wt. %)	100	90–100	68–85	38–68	24–50	15–38	10–28	7–20	5–15	4–8

**Table 3:** Technical Indicators of Chloride Salt.

Component							Density (g/cm^3^)	Solubility (g)
Items	NaCl	Water	Mg^+^	Ca^2+^	Water Insoluble Substance	Other Components		
Mass fraction (wt. %)	>99	<0.05	<0.01	<0.01	<0.05	<0.02	2.170	36

**Table 4:** Test Environment.

Environment	0%	5%	10%	15%	20%
Soak	AC (1)	AC (2)	AC (3)	AC (4)	AC (5)
Dry–wet cycle	AC (6)	AC (7)	AC (8)	AC (9)	AC (10)

**Table 5:** CT Scanning Parameters.

Scanning Voltage	Scanning Mode	Window Width	Window Position	Layer Thickness	Resolving Power
140 kV	Cross section	3500	1500	1.5 mm	0.06 mm

**Table 6:** Test Environment for Specimens’ CT Scanning.

Environment	Purified Water	20% Salt Solution
Soak	AC1	AC2
Dry–wet cycle	AC3	AC4

**Table 7:** CT Values of Each Part.

Asphalt Mortar	Aggregate	Water	5% Brine	10% Brine	15% Brine	20% Brine
998HU	3908HU	2HU	105HU	172HU	247HU	312HU

**Table 8:** Voidage Value and Calculated Voidage Value.

Voidage (%)	Test Value	T	T1
Initial AC1	4.5	4.59	6.73
Initial AC2	4.1	4.24	6.47
Initial AC3	4	4.03	6.52
Initial AC4	4.1	4.25	6.61

**Table 9:** The Voidage Value’s Calculated Relative Error.

Relative Error Value (%)	Test Value	T	T1
Initial AC1	0	1.98	49.56
Initial AC2	0	3.32	57.81
Initial AC3	0	0.85	63.00
Initial AC4	0	3.59	61.22

**Table 10:** Void Calculation Data for the AC1 Specimen (Under Water Soaking Conditions).

Factor	Voidage (%)	Open Voidage (%)	Number of Voids	Number of Open Voids	Mean Void Volume (mm^3^)	Open Voids’ Mean Volume (mm^3^)
0 day	4.59	0.94	2948	364	7.95	13.12
7 days	4.61	0.97	2935	359	8.02	13.77
14 days	4.61	0.96	2911	356	8.09	13.82
21 days	4.62	0.98	2891	359	8.16	13.99
28 days	4.63	1.02	2883	354	8.19	14.73

**Table 11:** Void Calculation Data for the AC2 Specimen (Under Salt Water Soaking Conditions).

Factor	Voidage (%)	Open Voidage (%)	Number of Voids	Number of Open Voids	Mean Void Volume (mm^3^)	Open Voids’ Mean Volume (mm^3^)
0 day	4.236	0.932	3048	379	7.15	12.65
7 days	4.321	0.994	2829	368	7.87	13.91
14 days	4.356	1.011	2826	357	7.94	14.58
21 days	4.354	1.028	2805	350	8.00	15.13
28 days	4.358	1.033	2792	348	8.04	15.29

**Table 12:** Void Calculation Data for the AC3 specimen (Under Dry and Water Conditions).

Factor	Voidage (%)	Open Voidage (%)	Number of Voids	Number of Open Voids	Mean Void Volume (mm^3^)	Open Voids’ Mean Volume (mm^3^)
0 day	4.03	0.93	2724	313	7.58	15.23
7 days	4.09	0.97	2651	284	7.91	17.45
14 days	4.123	0.98	2615	278	8.08	18.09
21 days	4.13	0.10	2535	280	8.35	18.23
28 days	4.15	1.02	2471	275	8.60	19.09

**Table 13:** Void Calculation Data for the AC4 specimen (Brine under Dry and Wet Cycling Conditions).

Factor	Voidage (%)	Open Voidage (%)	Number of Voids	Number of Open Voids	Mean Void Volume (mm^3^)	Open Voids’ Mean Volume (mm^3^)
0 day	4.25	0.89	2767	334	7.94	13.80
7 days	4.52	1.07	2495	284	9.39	19.50
14 days	4.52	1.12	2437	274	9.61	21.19
21 days	4.51	1.13	2416	270	9.68	21.61
28 days	4.54	1.17	2166	260	10.88	23.29

## References

[1] Huang Y.X., Sha A.M., Jiang W., Wang Y.N. (2017). Effect of salt erosion on asphalt and mixture performance and its mechanism. J. Chang’an Univ..

[2] Setiadji B.H., Nahyo S.U. (2016). Effect of chemical compounds in tidal water on asphalt pavement mixture. Int. J. Pavement Res. Technol..

[3] Xiong R., Chen S.F., Guan B.W., Sheng Y.P. (2014). Study on low temperature cracking resistance of fiber asphalt mixture under sulfate-dry-wet cyclic erosion environment. J. Wuhan Univ. Technol..

[4] Chen S.F., Chen H.X. (2011). Effect of deicing salt on asphalt concrete performance. Highway.

[5] Zhang K., Zhang Z.Q. (2015). Degradation of mechanical properties of asphalt mixture in salty and humid environment. J. South China Univ. Technol..

[6] Shman L.S. (2001). Internal structure analysis of asphalt mixes to improve the simulation of superpave gyratory compaction to field conditions. Maine Law Rev..

[7] Xiong R., Chen S.F., Guan B.W., Li Z.Z., Zhao H. (2011). Durability of asphalt mixture under sulfate corrosion environment. J. Chang’an Univ..

[8] Wu Z.M., Gao P.W., Chen D.F., Zhang H.B., Cheng X., Ling Y.C. (2012). Effect of chloride salt snow melting agent on low temperature crack resistance of asphalt mixture. Highw. Eng..

[9] Ma Q.Y., Wu J.R., Qin K. (2013). Test and analysis of the influence of chloride salt on the tensile strength of freeze-thaw cracking of asphalt concrete. Ice River Frozen Soil.

[10] Qin K., Ma Q.Y., Wu J.R. (2013). Experimental study on the performance of asphalt concrete under the coupling of temperature and corrosion. Buffalo Bul..

[11] Zhou J.Z., Zheng J.H. (2011). Experimental study on low temperature performance of asphalt concrete under chloride salt attack. Chin. Foreign Highw..

[12] Cha X.D., Ren X., Fu G.W. (2012). Analysis of the effect of chloride salt snow melting agent on the road performance of SBS modified asphalt mixture. Transp. Sci. Eng..

[13] US Department of Transportation (1998). Simulation, Imaging and Mechanics of Asphalt Pavement.

[14] Masad E., Muhunthan B., Shashidhar N., Harman T. (1999). Quantifying laboratory compaction effects on the internal structure of asphalt concrete. Transp. Res. Rec..

[15] Masad E., Button J. (2004). Implications of experimental measurements and analyses of the internal structure of HMA. Transp. Res. Rec..

[16] Fu Y.R., Wang L.B., Tumay M.T., Li W. (2008). Quantificationand simulation of particle kinematics and local strainin granular materials using X-ray tomography imaging and discrete-element method. J. Eng. Mech..

[17] Wang L.B., Wang X., Mohammad L., Wang Y. (2004). Application of mixture theory in the evaluation of mechanical properties of asphalt concrete. J. Mater. Civ. Eng..

[18] You Z.P., Adhikari S., Dai Q.L. (2008). Three-dimensional discrete element model for asphalt mixtures. J. Eng. Mech. ASCE.

[19] Liu Y., You Z.P. (2009). Visualization and simulation of asphalt concrete with randomly generated three-dimensional models. J. Comput. Civ. Eng..

[20] Zhou C.X., Tan Y.Q. (2009). Influence factors of snow and ice removal performance of rubber granular asphalt mixture. J. Build. Mater..

[21] Zavrtanik N., Prosen J., Tušar M., Turk G. (2016). The use of artificial neural networks for modeling air void content in aggregate mixture. Autom. Constr..

[22] Masad E., Jandhyala V.K., Dasgupta N., Somadevan N., Shashidhar N. (2002). Characterization of air void distribution in asphalt mixes using X-ray computed tomography. J. Mater. Civ. Eng..

[23] Yu W.S., Zhang X.C., Zhong K. (2015). Deicing Characteristics of High Elastic Storage Salt Melting Ice and Snow Asphalt Mixture. J. China Univ. Min. Technol..

[24] Hu J., Qian Z., Liu Y., Zhang M. (2015). High-temperature failure in asphalt mixture using micro-structural investigation and image analysis. Constr. Build. Mater..

[25] Gao J., Guo H.Y., Wang X.F., Wang P., Wei Y., Wang Z., Huang Y., Yang B. (2019). Microwave Deicing for Asphalt Mixture Containing Steel Wool Fiber. J. Clean. Prod..

[26] He T., Cheng L.F., Wu Q.H., Wang Z.J., Yang L.G., Xie L.Y. (2015). An Image Segmentation Calculation Based on Differential Box-Counting of Fractal Geometry. Appl. Mech. Mater..

[27] Zhou X.L., Xiao S.Q., Ran M.P. (2016). Asphalt pavement aggregate separation evaluation method based on multifractal theory. J. Wuhan Univ. Sci. Technol..

[28] Yu J., Zhang F. (2015). Sandbox Method for Void Fractal Dimension of Asphalt Mixture. J. Highw. Eng..

[29] Xiong R., Chu C., Qiao N., Wang L., Yang F., Sheng Y., Guan B., Niu D., Geng J., Chen H. (2019). Performance evaluation of asphalt mixture exposed to dynamic water and chlorine salt erosion. Constr. Build. Mater..

[30] Golewski G.L. (2017). Determination of fracture toughness in concretes containing siliceous fly ash during mode III loading. Struct. Eng. Mech..

[31] Sadowski T., Golewski G.L. (2018). A failure analysis of concrete composites incorporating fly ash during torsional loading. Compos. Struct..

